# Data-Driven Anomaly Detection in High-Voltage Transformer Bushings with LSTM Auto-Encoder

**DOI:** 10.3390/s21217426

**Published:** 2021-11-08

**Authors:** Imene Mitiche, Tony McGrail, Philip Boreham, Alan Nesbitt, Gordon Morison

**Affiliations:** 1 Department of Computing, School of Computing, Engineering and Built Environment, Glasgow Caledonian University, Glasgow G4 0BA, UK; Gordon.Morison@gcu.ac.uk; 2 Doble Engineering, Bere Regis BH20 7LA, UK; tmgrail@doble.com (T.M.); pboreham@doble.com (P.B.); 3 Department of Electrical and Electronic Engineering, School of Computing, Engineering and Built Environment, Glasgow Caledonian University, Glasgow G4 0BA, UK; a.nesbitt@gcu.ac.uk

**Keywords:** transformer bushings, insulation failure, anomaly detection, LSTM, auto-encoder

## Abstract

The reliability and health of bushings in high-voltage (HV) power transformers is essential in the power supply industry, as any unexpected failure can cause power outage leading to heavy financial losses. The challenge is to identify the point at which insulation deterioration puts the bushing at an unacceptable risk of failure. By monitoring relevant measurements we can trace any change that occurs and may indicate an anomaly in the equipment’s condition. In this work we propose a machine-learning-based method for real-time anomaly detection in current magnitude and phase angle from three bushing taps. The proposed method is fast, self-supervised and flexible. It consists of a Long Short-Term Memory Auto-Encoder (LSTMAE) network which learns the normal current and phase measurements of the bushing and detects any point when these measurements change based on the Mean Absolute Error (MAE) metric evaluation. This approach was successfully evaluated using real-world data measured from HV transformer bushings where anomalous events have been identified.

## 1. Introduction

In high-voltage (HV) power systems a bushing is an electrical insulator that allows an electrical conductor to pass safely through a conducting barrier such as the tank of a power transformer [1]. Previous transformer failure and explosion incidents, about 12% in [2] and 40% cited in [3], were found to be related to bushing breakdown in power transformers. Such failures often come with heavy financial consequences, therefore, continuous monitoring of bushing condition is justified. The latter can be achieved by monitoring the leakage current measured at the individual bushing test tap [4,5]. There exist other measurement types to assess bushing condition such as power factor [6], capacitance, dissipation factor and partial discharges [7]. The analysis involves monitoring the measurement’s trend, such that when a certain value or percentage is reached, the bushing is diagnosed to be in a state of deterioration. Few bushing monitoring methods that analyse leakage current using Machine Learning (ML) methods have been proposed in the literature. In [8], an artificial neural network was trained in a supervised learning manner where the measured amplitude and phase were used as inputs and the bushing condition as an output or label. However, this is a supervised classification task of different bushing conditions. Anomaly detection in bushing leakage current using ML techniques has not been investigated in the literature, to the authors’ best knowledge. To fill this gap, we develop a data analysis method based on self-supervised ML that monitors changes in bushing leakage current and phase angle measurements in real-time. The method aims at identifying unexpected changes in the expected behaviour of a bushing system.

The measurements investigated in this paper consist of real-world leakage current magnitude and phase angle collected at three bushing taps over time. Due to the time-series nature of the data, we propose a method based on the state-of-the-art Long Short-Term Memory (LSTM) network [9] in an Auto-Encoder [10] framework that we call LSTMAE. LSTM is derived from Recurrent Neural Network (RNN) [11] and the goal behind using LSTM is to exploit its characteristic of keeping information on previous events to make the next decision, and this makes it useful in anomaly detection problems. The proposed method involves training the LSTMAE model on normal operation measurements as inputs in a self-supervised way, where the decoder part of the Auto-Encoder attempts to reconstruct and output a signal that is as close as possible to the input signal. The similarity between the original and reconstructed values is evaluated with the Mean Absolute Error (MAE) metric. The latter is also exploited in the anomaly identification part of the proposed analysis method, where it is compared to a threshold derived from the normal operation measurements.

Surveys on anomaly detection in time-series data were conducted in [12,13]. The authors covered a number of methods that address univariate and multivariate data types that were categorised as: statistical, pattern matching, distance-based, clustering-based and probabilistic predictive methods. We compare our work to methods that fall under the aforementioned categories except for pattern matching as this type of methods learn from both anomalous and normal data in a supervised approach, however, the data available in this work address a different scope. Two distance-based and clustering-based methods known as the Local Outlier Factor (LOF) [14] and Natural Outlier Factor (NOF) [15] are evaluated. For the probabilistic predictive category the autoregression-based methods are popular in anomaly detection [12]. We further implement Seasonal Autoregressive Integrated Moving Average (SARIMA) [16] and Vector Autoregressive (VAR) [17] models. For the statistical category we apply the simple moving average method calculated on the time series.

The remainder of this paper is structured as follows. The next section provides an overview on HV bushing with more insight on bushing condition monitoring. Section 3 describes the analysis method blocks used in this work, including RNN, LSTM and Auto-Encoder networks, and how they are combined to produce the LSTMAE architecture along with the anomaly identification steps. Section 4 describes the data and experiments investigated in this paper and shows the experiment’s results along with discussions and remarks on the presented work. Finally, the last section provides a summary and conclusions to this work with future recommendations.

## 2. High-Voltage Bushing Overview

A bushing provides a means for a conductor, usually high-voltage, to pass through a barrier, which is usually grounded. The bushing provides insulation between the conductor and the barrier. The insulation of the bushing often features stress-grading foils to ‘even out’ the voltage stress [18]. Bushing insulation provides a capacitive path to ground, and is not perfect, so there is a leakage current to ground through the bushing. If the insulation deteriorates, the current will change, usually rising: offline testing is very effective at detecting such deterioration, evaluating bushing power factor (tangent delta) and capacitance [19]. Online monitoring to detect incipient bushing failures really became popular in the 1990s. Originally, the idea was that a balanced system (same Root Mean Square (RMS) per phase at $120^{\circ }$ difference) with identical bushings would have the three bushing leakage currents sum to zero [20]; this proved disappointing as the system is not always balanced and there were too many false positives. As a result, the monitoring migrated to looking at leakage current magnitudes and phase for each bushing. At the time, readings of each value were taken hourly as the failure modes were suspected of being relatively ‘graceful’ with deterioration being evident over weeks to months [21]. The actual process in successful monitors was to record leakage current waveforms and parse those to extract the RMS value and relative phase between bushings; these values could then be used to derive the power factor and capacitance of each bushing [22]. These were important steps as power factor and capacitance are stamped on the nameplate for the bushing and can be used to directly identify deterioration, which is a significant benefit for field staff who are comfortable with the power factor and capacitance, and there are standards and guides available to provide acceptable limits on individual bushings. However, it must be noted that the current RMS and phase may vary rapidly, and vary inconsistently between phases, and provide RMS changes at a rate of up to 1.5% in a quarter of an hour. Consequently, the derivation of power factor and capacitance are based on a moving average, usually calculated over a day, a week and a month [23]. It is recommended that where a power factor or capacitance indicates deterioration, the current and phase data behind those values are investigated, as should be the original raw data of the waveforms recorded. The result of the change to averaged individual bushing values was a number of saves of a variety of different bushing types and designs: ABB, Westinghouse, PCore, GE, to name a few, which were confirmed ‘bad’ through subsequent offline tests. However, the advent of a rise in field failures of Trench COT and COTA bushings in the mid to late 2000s lead to an identified failure mode which was much more rapid—two saves using the standard hourly measurements with Trench bushings in Australia showed that the failure mode related to rapid deterioration of the insulation at the edges of the stress grading foils—a domino effect as once one foil is lost by being burned through or punctured, there is increased stress across the remaining insulation, which can accelerate the process. The two saves in 2012 showed the nominal current rising by almost 50% in 2 h, with concurrent rises in the power factor and capacitance. Subsequent to this, increased sampling is used for bushings where a rapid failure mode may occur—in fact, a number of users prefer a sampling rate of 5 min or 15 min. In addition, alerts may be set based on daily weekly and monthly variations in power factor and capacitance values, but also directly on current magnitudes and phase angles. The system in this article was installed in 2008 on the six low-voltage bushings of an externally completed ungrounded delta winding; readings were taken hourly on each bushing as was standard for systems manufactured at that time. The owners of the bushing (and the transformer and the associated generator) were and are very familiar with power factor and capacitance, and relied on those as their main point of focus for alert generation; they do not usually look at the raw wave forms or current/phase values. In addition, they rely on the monitor supplier to provide advanced technical support—data interpretation, diagnostics and prognoses.

## 3. Materials and Methods

A description of RNN and LSTM networks is first provided in this section, then details on how LSTM is incorporated in an Auto-Encoder along with anomaly detection algorithm steps are discussed.

### 3.1. Recurrent Neural Network (RNN)

**Figure 1:**
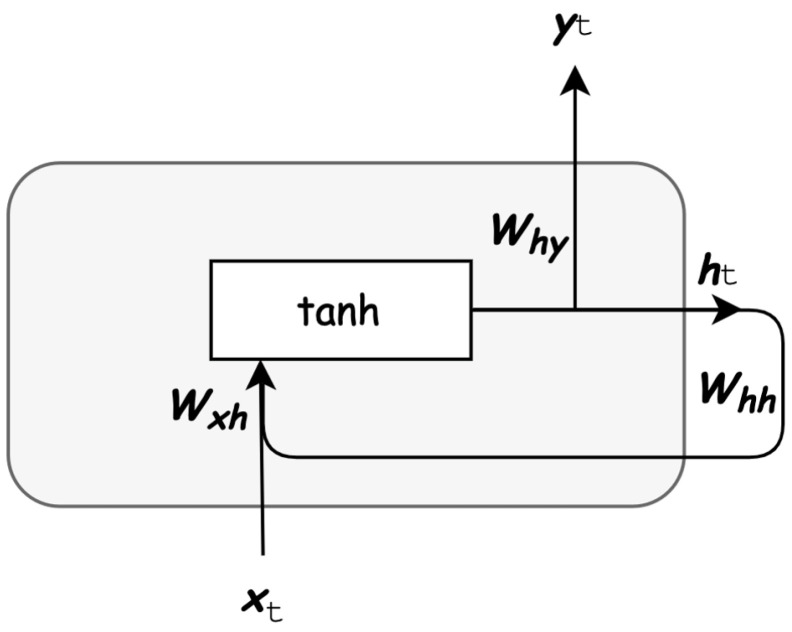
Recurrent Neural Network block.

RNN basic architecture can be seen as a looped block, also referred to as the repeating module, or multiple blocks connected in series, where an input observation vector $x_{t}$ passes through the block consisting of a hidden vector $h_{t}$ as

(1) $$h_{t}=H\left(W_{\mathrm{xh}}x_{t}+W_{\mathrm{hh}}h_{t-1}+b_{h}\right)$$

where $W_{\mathrm{xh}}$ is the input-hidden weight matrix, $W_{\mathrm{hh}}$ is the hidden-hidden weight matrix, and $b_{h}$ is the hidden bias vector, and H represents an element-wise operation, e.g., a tanh or sigmoid function. Thus the RNN block consists of a single layer. The block produces an output value $y_{t}$ at time point t=0,1,2,…N with *N* being the time-series length, as shown in Equation (2).

(2) $$y_{t}=W_{\mathrm{hy}}h_{t}+b_{y}$$

with $W_{\mathrm{hy}}$ being the hidden-output weight matrix. This shows that in RNN each block passes a message to the next one, making this architecture suitable for sequences of data, see Figure 1.

Long-term dependency of information is important in some cases where further past information is required to make a better prediction. It was found in [24] that in practice RNNs are incapable of learning the long-term dependencies, however, this can be achieved by the special case of RNN which is LSTM.

### 3.2. Long Short-Term Memory (LSTM)

**Figure 2:**
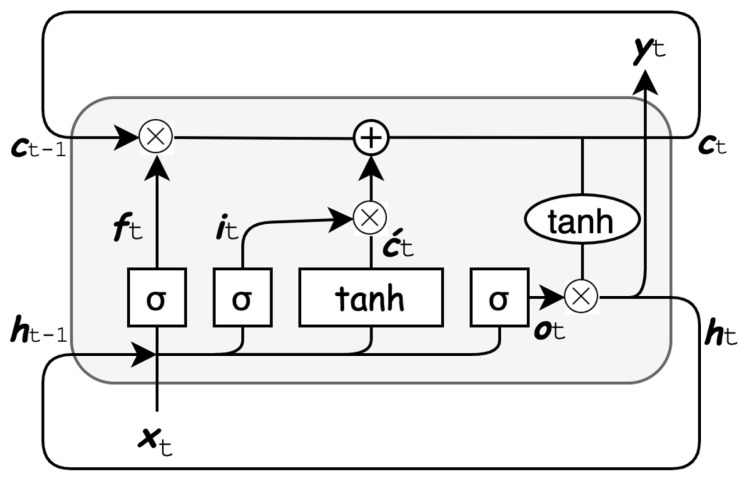
Long Short-Term Memory block.

LSTM was first introduced by [9] and has been widely used in various problems such as speech recognition [25]. Its main functionality is to hold information for a longer period of time. LSTM architecture is similar to RNN’s except that LSTM’s block consists of multiple interconnected layers, along with the pointwise addition and multiplication operations which play an important role in adding or removing relevant information from the block. The LSTM composition used in this paper is illustrated in Figure 2 and is defined as:

(3) $$i_{t}=\sigma \left(W_{\mathrm{xi}}\cdot \left[h_{t-1},x_{t}\right]+b_{i}\right)$$

(4) $$f_{t}=\sigma \left(W_{\mathrm{xf}}\cdot \left[h_{t-1},x_{t}\right]+b_{f}\right)$$

(5) $${\overset{‵}{c}}_{t}=tanh\left(W_{\mathrm{xc}}\cdot \left[h_{t-1},x_{t}\right]+b_{c}\right)$$

(6) $$o_{t}=\sigma \left(W_{\mathrm{xo}}\cdot \left[h_{t-1},x_{t}\right]+b_{o}\right)$$

(7) $$h_{t}=o_{t}\times tanh\left(c_{t}\right)$$

where $i_{t},f_{t},{\overset{‵}{c}}_{t},o_{t}$ are the inputgate, forgetgate, candidategate and outputgate, respectively. The main characteristic of LSTM block is the top horizontal line within the LSTM block, as shown in Figure 2. The forgetgate decides which information is no longer relevant and needs to get discarded. The candidategate, on the other hand, decides which information should be added to the cellstate $c_{t}$. The final output is produced by the output gate multiplied by the cell state that is passed to a tanh function to obtain values between −1 and 1. The previous cell state $c_{t-1}$ is updated using (8) to obtain the new state $c_{t}$.

(8) $$c_{t}=f_{t}\times c_{t-1}+i_{t}\times {\overset{‵}{c}}_{t}$$

### 3.3. Long Short-Term Memory Auto-Encoder (LSTMAE)

In neural networks, Auto-Encoders [26] can be split into two main functions. The first one is the encoder with weights $W_{e}$ which maps the input x into a latent feature space F, as defined in Equation (9). The second part is the decoder with weights $W_{d}$ which maps the latent feature space into a reconstructed version of the input, denoted as $\hat{x}$ and defined in Equation (10), where ϕ is an activation function.

(9) $$F=\phi (W_{e}x)$$

(10) $$\hat{x}=W_{d}F=W_{d}\phi \left(W_{e}x\right)$$

**Figure 3:**
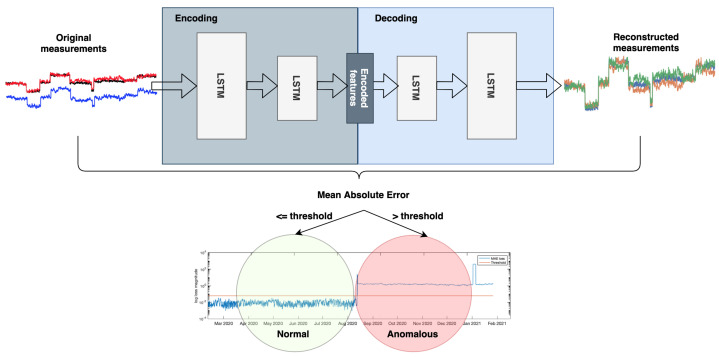
Long Short-Term Memory Auto–-Encoder Network (LSTMAE).

The LSTMAE model architecture used in this paper is illustrated in Figure 3, each of the encoder and decoder contains two LSTM blocks. The input to LSTMAE is designed to input a batch of size = 10 by 1 time stamp by 3 features. The latter correspond to three measurement signals from each tap of the bushing, therefore, the model can be considered as multivariate. The model was trained over 100 epochs using the ADAM optimizer [27] to reduce the Mean Absolute Error (MAE) loss, defined in Equation (11). Of the total training data, 5% were reserved for validation after each epoch. The model was implemented and trained in an Anaconda environment using Keras interface with Tensorflow backend. No GPUs were required for training the model, instead, it was performed on a Mac OS with 2.2 GHz Quad-Core Intel Core i7 CPU and 16 GB memory.

(11) $$L=|x-\hat{x}|$$

The aim behind training the LSTMAE in this work is to learn measurements in normal operation through encoding them into meaningful features during the encoding part. The decoder then attempts to replicate the normal measurements. The MAE loss, defined in Equation (11) is calculated between the original measurement $x_{t}$ and its reconstructed version ${\hat{x}}_{t}$, and is used to evaluate the LSTMAE learning in a way that it is minimized so the reconstructed measurements are as close as possible to the original measurements.

### 3.4. Anomalous Event Decision

The algorithm for anomaly detection can be partitioned in two parts. The first part is the learning of data under normal condition, this is achieved as follows:

1. Train the LSTMAE model by reducing the MAE loss;
2. Once the training has converged, calculate the MAE loss for each time step and fit to a distribution;
3. Derive the threshold for normal/anomaly as the boundary of the MAE score distribution calculated on the training data.

The second step is the anomaly detection part where the trained model is used as follows:

1. Input the time series to the LSTMAE model and derive the reconstructed version from the decoder;
2. Calculate the MAE between the original input time series and its reconstructed version;
3. Compare MAE score with the derived threshold for anomaly detection.

The theory behind using the MAE score as comparison between the original and reconstructed time series for anomaly detection is that after the encoder has successfully learned feature representation of the normal condition time-series, when an anomalous value is inputted to the LSTMAE, the model maps this input to the normal features resulting in reconstructed value closer to the normal condition value, therefore, the MAE score is expected to be higher than the threshold. This is observed and discussed further in the Section 4.

## 4. Results and Discussion

First, a description of the data measurement and apparatus is provided. Two analysis experiments using for LSTMAE model are conducted, and their respective results are presented and discussed. Results from other anomaly and outlier detection methods are also presented and discussed.

### 4.1. Bushing Data Measurement

**Figure 4:**
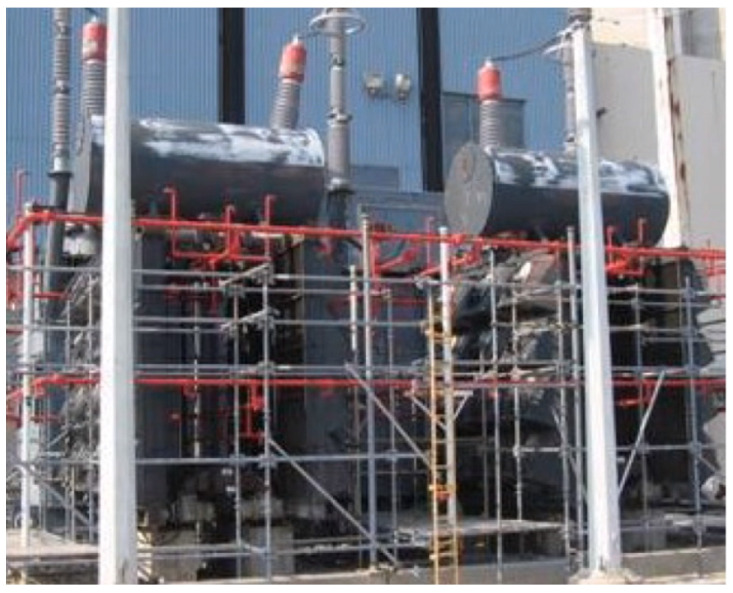
Site operating GE 980MVA Step-Up transformer.

**Figure 5:**
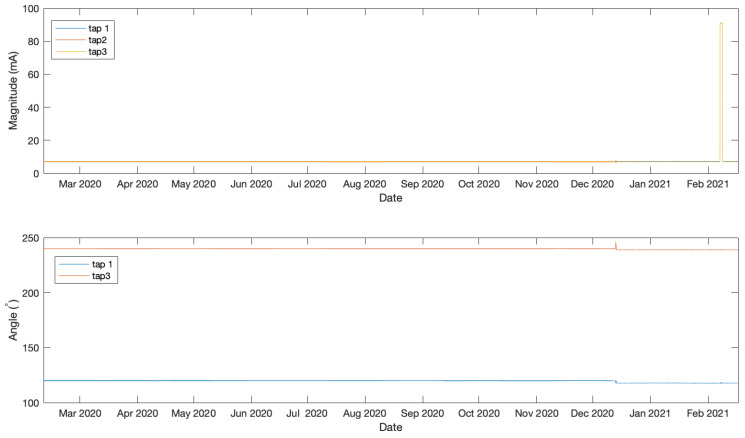
Angle and magnitude data.

The data analysed in this work were measured from taps of three bushings belonging to a 1972 two-winding Generator Step-Up 980 MVA transformer manufactured by General Electric, which is shown in Figure 4. The transformer’s operation receives 345 V from a generator and outputs a voltage of 23.75 kV. The bushings under investigation in this work are connected to the low-voltage ends in a Wye–Delta configuration. Leakage current and phase angle were acquired every hour between 10 February 2020 at 12:00 and 17 February 2021 at 1:00 using a doblePRIME IDD Bushing Monitor device. The acquired data from the three bushings are plotted in Figure 5. Note that in this work we are only using phase angle data from Tap 1 and 3 as they are relative to Tap 2 which is constant and set to zero. The data underwent a normalisation between 0 and 1 as a processing step prior to being passed to the LSTMAE model.

### 4.2. Experiment 1

**Figure 6:**
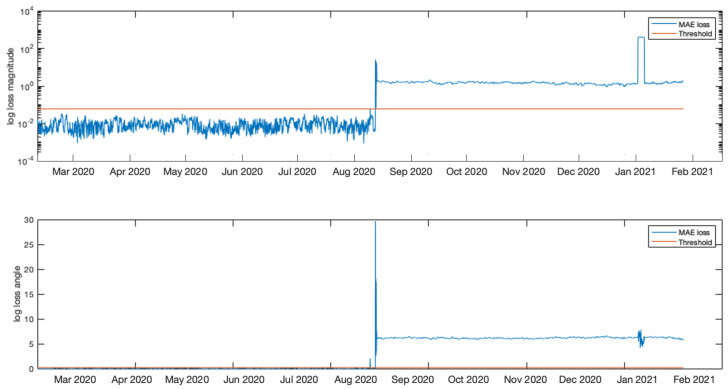
MAE loss and the derived threshold on the data after complete training.

**Figure 7:**
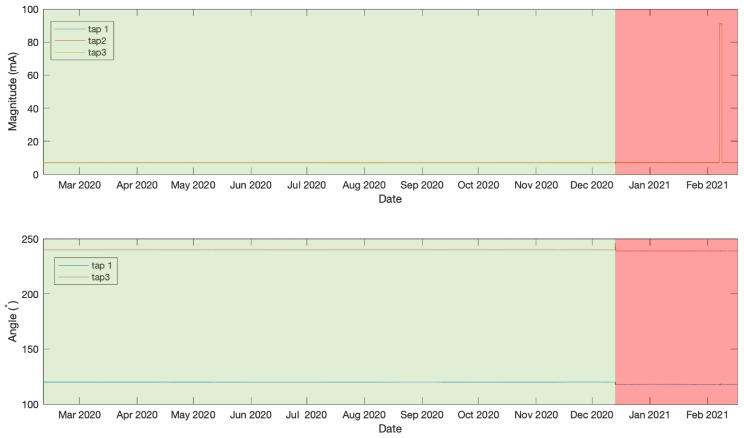
Anomaly detection result on magnitude and angle data in experiment 1.

**Figure 8:**
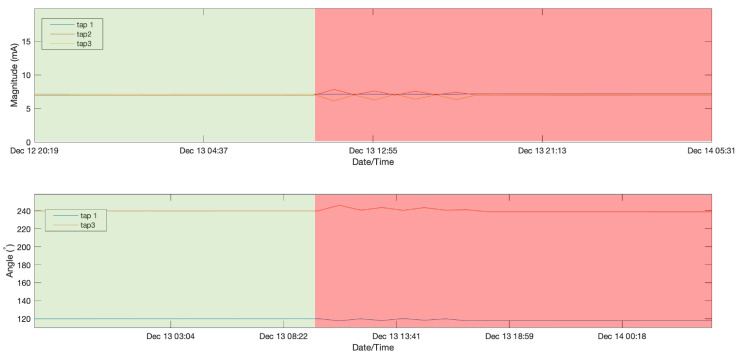
Magnified anomaly detection result on magnitude and angle data in experiment 1.

In this initial experiment the first days of data measurements were identified as a normal operation and therefore were used to train the LSTMAE model. This includes measurements from 10 February 2020 at 12:00 until 13 December 2020 at 18:00 and is illustrated in Figure 5. The remaining data measurements, beyond this point until 17 February 2021 at 1:00, contain both normal and anomalous events and were used to test the model. Figure 6 shows the MAE loss of the test data when evaluated using the LSTMAE model after training. It is observed that on 24 December 2020 at 20:00 the MAE increased above the anomaly decision threshold. The results can be observed in Figure 7 for magnitude and angle data, where the green shaded area is identified as normal and the red shaded area as anomalous. By looking at the overall signal, the difference beyond the measurement of day 24 December 2020 at 20:00 is not clear, however, when looking closer at the signal (see Figure 8), a slight increase is observed in Tap 1 and Tap 2 magnitudes and a decrease in tap 3 magnitude after few fluctuations in Tap 2 and Tap 3 magnitudes. Similar results are observed in phase angle data where the angle values fluctuate then decrease to a steady value. Another anomaly is observed few months later where very high values in magnitude were measured and a disruption in angle was also observed at the same time. After an investigation by online monitoring engineers, the following possible explanations were made. The anomaly could be related to shielding issues inside the transformer. Furthermore, the winding and bushing will be susceptible to sudden failure through switching, a fault or transient surges. It was also concluded that the fluctuations have stabilized, making the new leakage current and angle readings the new normal as the sum of the leakage current from the three taps before and after the anomalous event is the same.

### 4.3. Experiment 2

In this experiment we consider the point after the measurement fluctuations as the new normal since the values have been steady after that point without breakdown, as per online monitoring engineers’ suggestion. The main goal behind this experiment is to demonstrate that the model can be flexible and re-trained on later measurements that are considered as the new normal and a new threshold was derived for this set. The test data set from experiment 1 was split into another training and testing set on which the model was evaluated.

**Figure 9:**
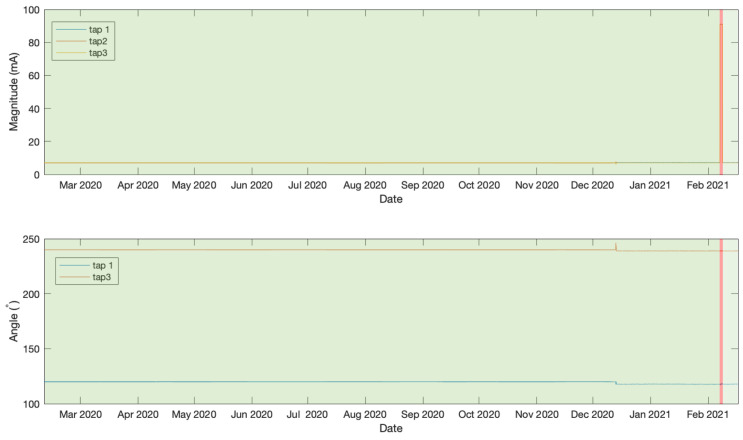
Anomaly detection result on magnitude and angle data in experiment 2.

Results are presented in Figure 9 for anomaly detection on current and angle data. An anomaly was detected in both measurements between 7 February 2021 at 10:00 and 8 February 2021 at 17:00. The online monitoring engineers justified the sudden surge in current and angle fluctuations as a data acquisition issue. Another anomaly was detected in the current measurement only on 15 February 2021 3:00. This is due to small current fluctuations and should be monitored closer in combination with angle measurements and look for a repetition rate, as a false positive event may occur and may not be an indication.

### 4.4. Comparison to Other Methods

We present results from five methods that have been previously used in anomaly detection as follows:

#### 4.4.1. Autoregressive Models

Both VAR and SARIMA use an autoregressive model but differ slightly in their parameters, more details on the algorithm can be found in [16,17]. VAR is more suitable for multivariate and endogenous time series, which is the nature of our dataset. However, SARIMA is more suitable for exogenous time series and supports univariate time series only. For comparison purposes, in this work we use several SARIMA models for each of the taps’ magnitude and angle data. This may not be ideal for edge implementation in terms of memory and computation compared to the other methods. Since VAR and SARIMA are predictive models, they are trained on normal events and predict the remaining of normal and anomalous time series which is the test set. A rolling error is then calculated between the actual test data values and the predicted values by each model. For anomaly detection, we follow the same threshold method which is derived from the normal rolling error boundaries.

**Figure 10:**
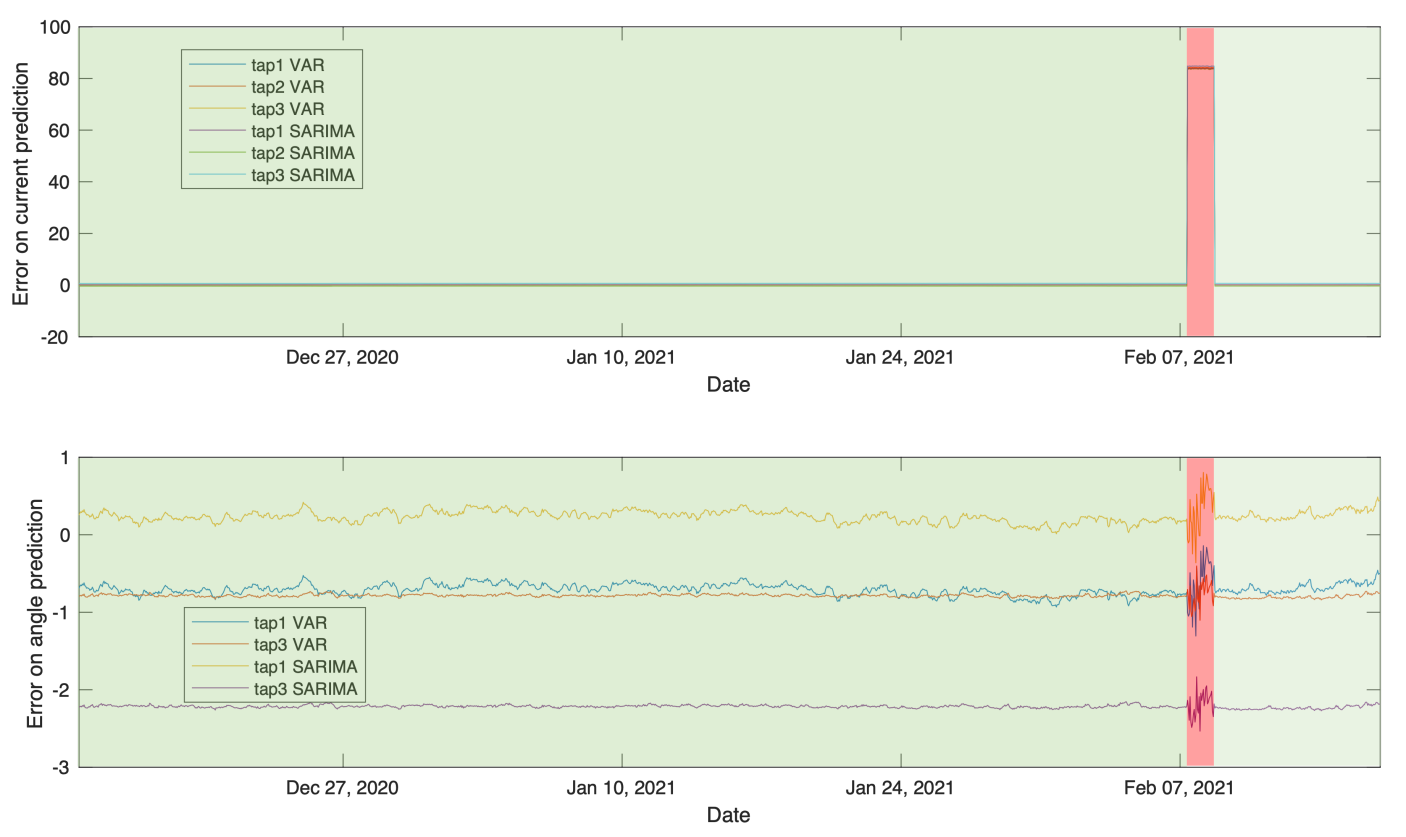
VAR and SARIMA rolling error between predicted and actual test data.

Figure 10 shows the rolling error results for both VAR and SARIMA models. It is observed that the second anomaly in February has successfully been identified, however, the first anomaly in December was undetectable. This is due to the rolling error being smaller than the threshold.

#### 4.4.2. Distance and Clustering Models

The LOF and NOF methods were founded on the concept of comparing the local density of a point with its neighbours, where the local density is calculated between the point and its k nearest neighbours. Therefore, these two methods were evaluated on the full available dataset. The anomaly or outlier points can be identified as points having lower density than their neighbours, whereas regions of similar density are considered as normal. For more details on the algorithm readers are referred to [14,15].

**Figure 11:**
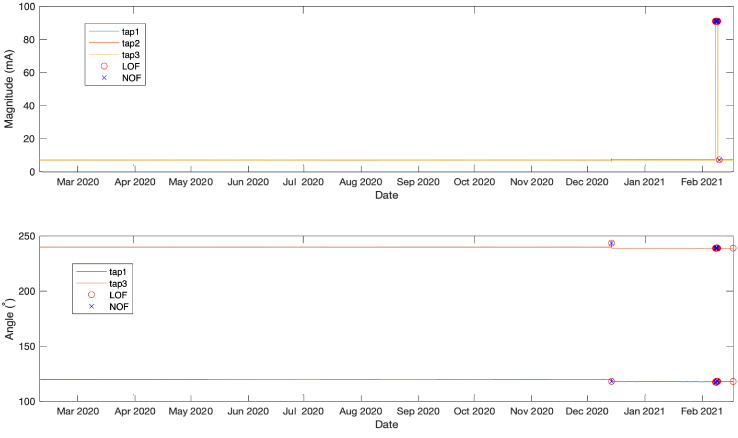
LOF and NOF outlier detection results.

It is observed from the results in Figure 11 that both methods identified the anomalies on angle data, with one false positive towards the end of the measurements identified by LOF. For the magnitude data, both methods failed to identify the first anomaly on the magnitude measurements. These results can be explained by the large difference in magnitude and the small angle difference between the first and second anomaly.

#### 4.4.3. Moving Average Method

**Figure 12:**
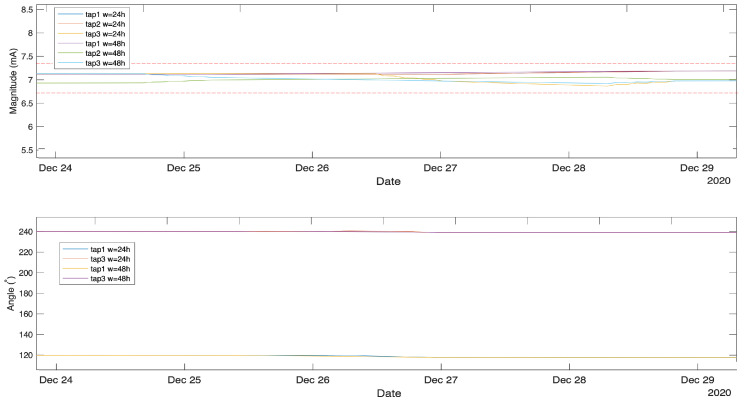
Moving average magnified at first anomaly date, dotted line represents the anomaly boundaries.

**Figure 13:**
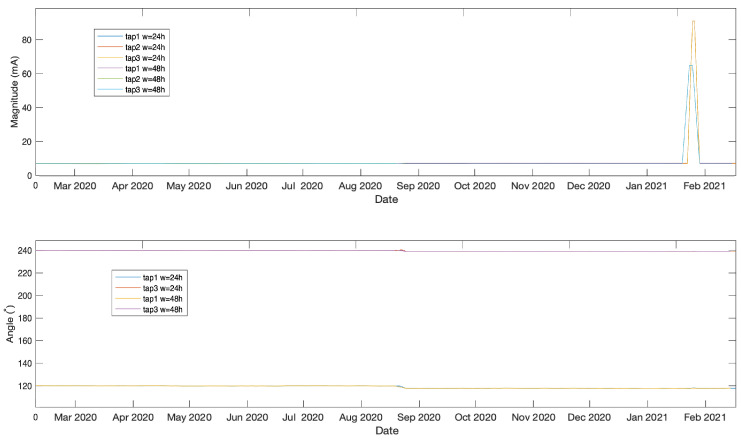
Moving average on the entire data set.

The moving average is one of the simplest methods that can be used in time-series anomaly detection which involves calculating the moving average of the historical data and using a standard deviation to find the boundary values which can define the anomalous points that fall beyond the boundaries. However, this technique is associated with the following drawbacks. First, the boundaries setting is sensible and may result in too many false positives due to moving average smoothing effect that results in a very small variance. If the boundaries are pushed further, it may result in undetected anomalies with small measurement variations, as seen in Figure 12. The second point is the slow response which is affected by the moving average time window of 24 or 48 h, see Figure 13 for the second anomaly. This makes the moving average method not ideal for a sudden change in measurement.

### 4.5. Further Remarks

The monitoring system will react quickly to changes–-the update rate for the bushing can be set to every few seconds, if need be, but if the suspicion is that a failure mode is available and in operation which is that fast, it would likely become an asset management decision to change out the bushing for one where such a failure mode is unlikely to occur. There is also a move towards continuous monitoring and linking the leakage current magnitude/phase to simultaneous extraction of transient and PD monitoring. Technically this is quite feasible, but the increased cost of such monitoring has to be weighed against the benefit—at present the in-service failure rate of bushings at distribution, transmission and generation sites around the world is usually less than 0.5% per year, resulting in a poor cost benefit, especially as the cause of many failures external to the bushing: animals, severe weather, vandalism, etc. Therefore, we can react to changes, sometimes a swing of >1.5% in 15 min, with a rate of change at 6% per hour is just a change in tap position on the transformer coupled with system loading variation. There are also interesting effects which can result from a transformer near a source of static var compensation or at the end of long overhead lines; the imbalance in MW/MVAr load can lead to variable phase angles resulting in anomalous power factor readings. Cooling the bushings will have a small effect—we do have tables for temperature correction of bushing measurements built into monitors, but the effect is usually small. An interesting effect is possible where pollution collects on the bushing surface, allowing for an alternate path to ground in the measurement system—the test object changes and the resulting power factor can reduce and become negative; a similar effect can be seen by the build-up of sludge, contaminants or moisture inside a bushing during operation. In particular, the ingress of moisture may lead to a rise in power factor, but if the moisture levels increase further within the bushing an alternate path to ground may be formed which allows for the power factor to fall and, in fact, turn negative. A little rain has very little effect in practice unless it can get inside the bushing—the case of rising/falling power factor was one where a bad gasket on a bushing fill plug was thought to be the cause, subsequently confirmed through offline tests and forensic tear-down of the bushing.

## 5. Conclusions

Anomalous events were successfully detected on leakage current and phase angle measured from three bushing taps that are part of a field operating transformer. Anomaly detection was achieved by LSTMAE model combined with MAE metric. Other anomaly detection methods were also investigated on our dataset where the methods failed to identify the first anomaly compared to the LSTMAE, particularly in the current measurements. It was also demonstrated that the model can be re-trained on new normal measurements that were previously identified as a change. To conclude, the proposed method is aimed at identifying any change in the measurement which indicates an anomaly and thus may indicate the onset of a fault in the bushing. Identification of a ‘new normal’ for the measurements does not, of itself, guarantee that there will be no new anomaly or deterioration. Our recommendation is to closely monitor the change duration and repetition rate and develop an action plan to respond to monitoring levels: caution, warning and action.

## Data Availability

The data used in this research is private due to client confidentiality policy of Doble Engineering Company.

## Abbreviations

The following abbreviations are used in this manuscript:

HV	High-Voltage
LOF	Local Outlier Factor
LSTM	Long Short-Term Memory
LSTMAE	Long Short-Term Memory Auto-Encoder
MAE	Mean Absolute Error
ML	Machine Learning
NOF	Natural Outlier Function
RMS	Root Mean Square
RNN	Recurrent Neural Network
SARIMA	Seasonal Autoregressive Integrated Moving Average
VAR	Vector Autoregressive
